# Discovery and functional study of lncRNAs associated with fat deposition in Kele pigs based on whole-transcriptome RNA sequencing

**DOI:** 10.5713/ab.24.0900

**Published:** 2025-04-28

**Authors:** Xiong Zhang, Yong Guo, Zhonglong Zhao, Tiansong Wang, Qingshi Wang, Runqian Yang, Zhaobi Ai, Yong Zhang

**Affiliations:** 1 Key Laboratory of Animal Genetics, Breeding and Reproduction in the Plateau Mountainous Region, Ministry of Education, College of Animal Sciences, Guizhou University, Guiyang, China; 2 Institute of Animal Husbandry and Veterinary, Guizhou Academy of Agricultural Science, Guiyang, China; 3 Guizhou Cattle Industry Group Science & Technology Services Co., Ltd., Guiyang, China; 4 Agricultural College, Tongren Polytechnic College, Tongren, China; 5 Guiyang Rural Revitalization Service Center, Guiyang, China

**Keywords:** Fat Deposition, lncRNA, Kele Pigs, Subcutaneous Fat Precursor Cells

## Abstract

**Objective:**

This study was conducted to identify lncRNAs associated with fat deposition in Kele pigs and investigate their molecular regulatory mechanisms.

**Methods:**

Six healthy 10-month-old male Kele pigs were selected as the experimental subjects and divided into high backfat thickness (>5%, H) and low backfat thickness group (<5%, L). Subcutaneous adipose tissue of each group was collected for whole-transcriptome RNA sequencing (RNA-seq). Subsequently, the biological functions of identified lncRNAs were investigated to explore their regulatory mechanisms on fat deposition in Kele pigs.

**Results:**

RNA-seq revealed 493 differentially expressed lncRNAs (p<0.05), with *TCONS_00185937* and *TCONS_00161198* implicated in fat deposition. Correlation analysis showed that the expression of *TCONS_00185937* was significantly negatively correlated with backfat thickness, and the expression of *TCONS_00161198* was significantly positively correlated with backfat thickness, and negatively correlated with eye muscle area. The pEGFP-*TCONS_00185937* was transfected into subcutaneous fat precursor cells, it was found that the expression levels of *TCONS_00185937* and its target genes *MOGAT2* and *ATGL* were significantly higher than control group (p<0.05). Similarly, pEGFP-*TCONS_00161198* transfected cells found that the expression levels of *TCONS_00161198* and its target genes *CSF3R* and *ACC* were significantly higher than control group (p<0.05). The induced differentiation of transfected cells found that the OD510 absorption value of pEGFP-*TCONS_00161198* group was higher than that of the control group. Triglyceride content was significantly higher than that of control group (p<0.01), while OD510 absorption value and triglyceride content of pEGFP-TCONS_00185937 group were lower than that of control group.

**Conclusion:**

The above results indicate that *TCONS_00161198* and *TCONS_00185937* may have regulatory effects on their target genes, thereby affecting fat deposition in pig subcutaneous fat precursor cells. This work provides a theoretical basis for the regulation of fat deposition in Kele pigs.

## INTRODUCTION

Animal body fat is mainly divided into subcutaneous fat and visceral fat, and subcutaneous fat thickness is also known as backfat thickness, which is closely related to genetic background, developmental stage and nutritional conditions. As an important economic livestock animal, pigs provide abundant animal protein for human beings [1]. Kele pig is a unique and valuable local pig breed in Guizhou Province, China, which has long adapted to the humid and hot climate at high altitude, formed a unique fat metabolism regulation mechanism, and is famous for its excellent meat quality and strong adaptability [2]. Compared with Large White pigs, Kele pigs have significant fat deposition characteristics, such as higher intramuscular fat content and superior meat flavor [3]. However, studies have shown that there were some problems such as slow growth rate and excessive subcutaneous fat deposition in Chinese local pig breeds [4]. Fat deposition occurred in the later stages of animal growth and development, mainly due to the coordination of fat decomposition and synthesis. Excessive fat deposition of subcutaneous fat in pigs will not only affect the taste of meat, but also reduce the feed conversion rate, thus restricting the breeding efficiency [5]. Due to differences in genetic background and environmental adaptation, subcutaneous fat deposition is usually more significant in Kele pigs. Therefore, this study focused on the regulatory effect of lncRNA on subcutaneous fat and explored its potential regulatory mechanism in Kele pigs.

LncRNA is a non-coding RNA molecule larger than 200 bp but lacking protein coding ability, which plays a role in epigenetic regulation, cell development and lipid synthesis in livestock and poultry [6–8], and can regulate mRNA expression at the posttranscriptional and transcriptional levels [9]. It was found that steroid receptor RNA activator 1 (*SRA1*) was the first lncRNA associated with adipocyte development [10]. RNA-seq was performed on the precursor cells of subcutaneous fat of Erhualian pigs at different stages of differentiation. The co-expression network analysis showed that lncRNAs *MSTRG.131380* and *MSTRG.62128* were related to adipogenesis, indicating that lncRNAs could affect the deposition of subcutaneous fat during the differentiation of pig adipocytes [11]. When RNA-seq was performed on the liver, muscle and adipose tissue of fatty Luchuan pig and lean Duroc, a total of 386/349/336 lncRNAs were screened, among which, 275 differentially expressed lncRNAs were located at 13 loci related to quantitative traits of fat deposition, which provided a basis for predicting lncRNA target genes by using the QTL_ID region [12]. Differential expression of lncRNAs, miRNAs, and mRNA in subcutaneous and intramuscular adipose tissue of three Laiwu female pigs were identified by deep RNA-seq, and it was found that *TCONS_006525*, *TCONS_0046551* and *TCONS_00000528* may be targeted to participate in Wnt signaling pathway, which plays a key regulatory role in intramuscular adipogenesis and lipid accumulation in Laiwu pigs [8]. In conclusion, lncRNAs play an important role in the regulation of fat deposition in pigs during development.

Many studies have shown that lncRNAs may regulate fat deposition through interaction networks with miRNA-lncRNA and lncRNA-mRNA. The mutual regulatory mechanism between lncRNA and miRNA includes lncRNA as a precursor of miRNA, competing with miRNA to bind mRNA to form a “sponge effect” mechanism [13]. Research has shown that the interaction between lncRNA and miRNA plays an important role in fat deposition. Specifically, different types of adipose tissue (such as brown adipose tissue and white adipose tissue) have differences in metabolism and function, which may lead to variations in the expression and function of lncRNA and miRNA [14]. Differential expression of lncRNA has been studied in adipose tissue of animals such as mice [15], sheep [16,17], and pigs [18]. lncRNA differentially expressed in the liver of diabetic mice and normal mice was compared by gene chip, and some lncRNAs with significant differentially expressed were identified. It was found that lncRNA *AK012226* had a regulatory effect on lipid accumulation in the liver of mice [15]. The differential expression of lncRNA in tail tissues of fat-tailed (Lori-Bakhtiari) and thin-tailed (Zel) was analyzed by comparative transcriptomes, and it was found that lncRNA may regulate the expression of genes related to lipid metabolism through cis-or trans-action [19].

Backfat thickness has always been an important economic trait in pig breeding, and the development of molecular technology has played a crucial role in the pig breeding. In this study, Kele pigs were selected as the experimental material, subcutaneous adipose tissue of Kele pigs with high and low backfat thickness groups was collected for RNA-seq, and lncRNA differentially expressed related to fat deposition was screened, and the main functions of lncRNA were predicted by functional enrichment analysis (GO/KEGG) of lncRNA and its target genes. Correlation studies were used to analyze the relationship between lncRNA and its target genes and backfat thickness traits, and to explore the effects of lncRNA on fat deposition at the cellular level. The purpose of this study was to provide a theoretical foundation for understanding the genetic regulation of subcutaneous fat deposition in Kele pigs, which may contribute to the future development of molecular breeding strategies.

## MATERIALS AND METHODS

### Animal care and samples collection

All procedures and animal care were authorized by the Institutional Animal Care and Use Committee of Guizhou University, Guiyang, China (EAE-GZU-2021-T113). The trial was carried out in strict accordance with relevant guidelines and regulations. Six healthy 10-month-old male Kele pigs, provided by Guizhou Younonggu Ecological Industry, were raised under identical dietary and management conditions. After slaughter, carcass and meat quality traits were measured. The screening threshold was 5% of average backfat thickness, the Kele pigs were divided into two groups: low backfat thickness group (<5%, L) and high backfat thickness group (>5%, H). Subcutaneous adipose tissues were collected and quickly stored in liquid nitrogen for later use.

### RNA extraction and genome sequencing

Total RNA was extracted with Trizol reagent, RNA concentration and purity were detected by Nanodrop spectrophotometer, and RNA integrity was accurately detected by Agilent 2100 bioanalyzer. Subsequently, the library was initially quantified by Qubit and diluted to 1 ng/μL, high-throughput sequencing was performed according to the effective concentration of the library and data production requirements (Illumina, PE150). The whole process is entrusted to Beijing Novogene Co.,Ltd. The RNA was reverse transcribed with reference to the reverse transcription kit (Supplement 1). The quantified cDNA was stored at −20°C for later use.

### Quality control, lncRNA screening and enrichment analysis

Fastp software was used for quality assessment and quality control of Raw reads. Clean reads were obtained after removing the Adapter sequence, reads with N ratio greater than 0.002 and low-quality reads (the number of bases with a mass value of Q ≤20 accounted for more than 50% of the entire read). The filtered valid data was mapped with *sus scrofa* Ensembl 97 reference genome using HiSAT2 software. Based on the comparison results, Stringtie was used to assemble transcripts, and the expression level of transcripts in each sample was calculated and standardized to FPKM. |log2FoldChange| >1, p<0.05 were used as screening criteria for differentially expressed lncRNAs. The main functions of lncRNA were predicted by functional enrichment analysis (GO/KEGG) of differentially expressed lncRNA target genes.

### Real-time quantitative polymerase chain reaction validation

To verify the accuracy of the results of RNA-Seq, 5 differentially expressed lncRNAs were randomly selected for reverse transcription quantitative polymerase chain reaction (RT-qPCR). Primer Premier 5.0 and NCBI BLAST software were used to design lncRNA fluorescent quantitative primers, and *GAPDH* was used as the reference gene (Supplement 2). The primers were synthesized by Shanghai Sangon Biotechnology. The reaction volume was 10 μL, 2×RealStar Green Fast Mixture with ROX 5 μL, forward primer 0.75 μL, reverse primer 0.75 μL, cDNA 1 μL and ddH_2_O 2.5 μL. The reaction procedure was pre-denaturation at 95°C for 5 min, denaturation at 94°C for 30 s, annealing and extension for 30 s, and 35 cycles.

### Construction of lncRNA expression vector and identification of bacterial solution and plasmid extraction

The full-length target fragments of 810 bp and 852 bp were amplified by PCR using the cDNA of subcutaneous adipose tissue. The sequence of the cloned primers and the gene primers related to fat deposition were shown (Supplement 3). Then the products were purified and connected with the vector. The reaction volume was 20 μL, including purified PCR product 5 μL, vector after enzyme digestion 5 μL and Mix 10 μL. After sequencing, the positive cloned bacterial solution was compared with the correct plasmid, and double enzyme digestion was performed with XhoI--HindIII polymerase. The 25 mL LB fluid nutrient medium was added with 60 μL of successfully connected and transformed bacterial solution and 25 μL ampicillin antibiotic, and the bacteria were shaken overnight at 37°C. The extraction of endotoxin-free plasmid was carried out according to the kit instructions.

### Cell transfection and induced differentiation

The cell suspension was inoculated in a 6-well plate and transfected 2 days after the cells were fully confluent according to the Lipofectamine 2000 reagent instructions. The transfected cell suspension was inoculated into the 6-well plate. 2 days after the cells were completely confluent, the medium was replaced with the induced differentiation medium, and this time was recorded as day 0. The cells were carefully cleaned with PBS containing double antibody before the medium was replaced, and the medium was replaced with the maintenance differentiation medium every 2 days after the induction.

### Extraction of total RNA in cells and cDNA synthesis

The culture medium in the culture plate was removed, cleaned with PBS for 3 times, 1 mL Trizol was added, and the cells were left for 5 minutes at normal temperature to fully lyse the cells. The cell RNA after transfection for 36 h was extracted, and the RNA was reverse-transcribed with reference to the reverse transcription kit, and the first strand of cDNA was synthesized for RT-qPCR.

### Oil red O staining and detection of triglycerides

On the 8th day of induced differentiation, after fixing the cells with 4% paraformaldehyde for 20–30 minutes, the cells were washed three times with PBS and stained with oil red O staining solution for 30 minutes. After discarding the oil red O working solution, the cells were cleaned with PBS for 3 times, and photographs were taken under an inverted fluorescence microscope. Equal amount of isopropyl alcohol was added into each cell hole and placed in a constant temperature shaking table at 37°C at 100 rpm/min. After 20 min, the absorption value of 510 nm wavelength was detected with a microplate reader.

### Statistical and bioinformatics analysis

The Online website (http://rna.informatik.uni-freiburg.de/IntaRNA/Input.jsp) was used to predict lncRNA and its target genes. The Relative gene expression levels were calculated by 2^−ΔΔCt^ method, using Excel 2016 to calculate data. The results were expressed as mean±standard deviation (M±SD). SPSS 20.0 software (IBM, Armonk, NY, USA) was used to conduct independent sample t-test to analyze the differences in gene expression levels between groups, and GraphPad Prism 8.0 software (GraphPad Software, San Diego, CA, USA) was used to create graphics.

## RESULTS

### Determination of carcass traits and meat quality indexes of Kele pigs

The carcass and meat quality traits of Kele pigs were determined (Table 1). In addition, we compared carcass quality traits of high and low backfat thickness groups and found significant differences in backfat thickness, eye muscle area, intramuscular fat content and skin thickness between the two groups (p<0.05) (Table 2).

### Quality assessment of RNA-seq data

**Figure 1:**
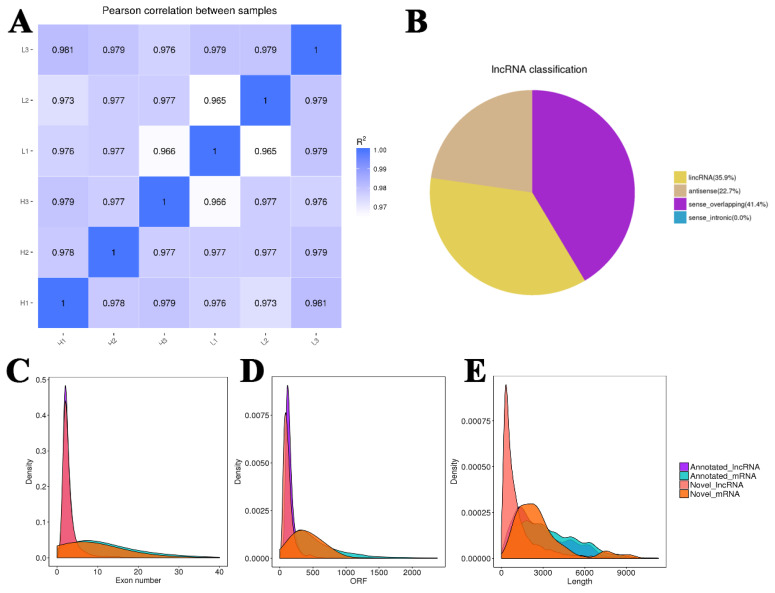
Analysis of RNA-seq data. (A) Inter-sample correlation tests. (B) Proportion of lncRNA types. (C) The number of exons of recognized and unrecognized mRNA/lncRNA. (D) ORF length of recognized and unrecognized mRNA/lncRNA. (E) Sequence length of recognized and unrecognized mRNA/lncRNA.

RNA-seq analysis of six samples from the high and low backfat thickness groups of Kele pigs showed that a total of approximately 525.4 million raw reads were generated from the 6 samples, with an average of 87,567,776 raw reads per sample. After filtering and quality assurance, about 507.9 million clean reads in total were acquired. The percentage of clean reads that were totally mapped to the pig reference genome varied from 84.36% to 93.50% (Table 3). The expression of three samples in each group was similar, and the sample repeatability was good (Figure 1A). 4,911 lncRNAs were newly discovered, with three types of sense overlapping (41.42%), lincRNA (35.88%) and antisense (22.70%) (Figure 1B). The exon number (Figure 1C), length (Figure 1D) and open reading frame (Figure 1E) were relatively concentrated. Most of the exon regions of lncRNA were concentrated within 5, and the length of newly discovered lncRNA was mostly concentrated within 2,000 bp.

### Screening and enrichment analysis for differentially expressed lncRNAs

**Figure 2:**
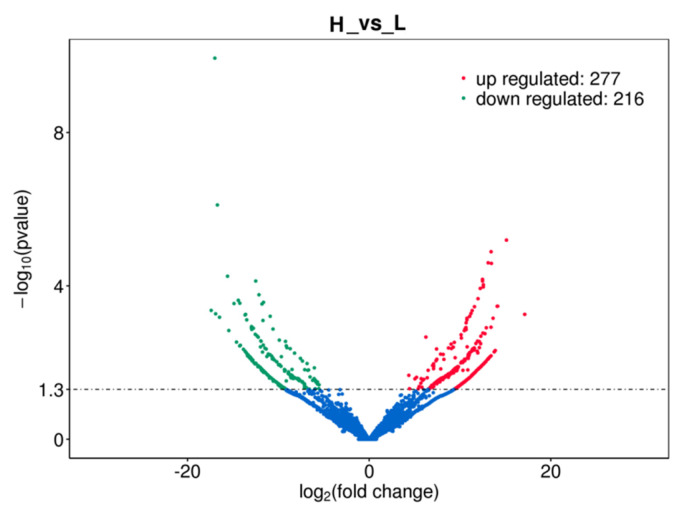
Volcano plot of differentially expressed lncRNAs.

**Figure 3:**
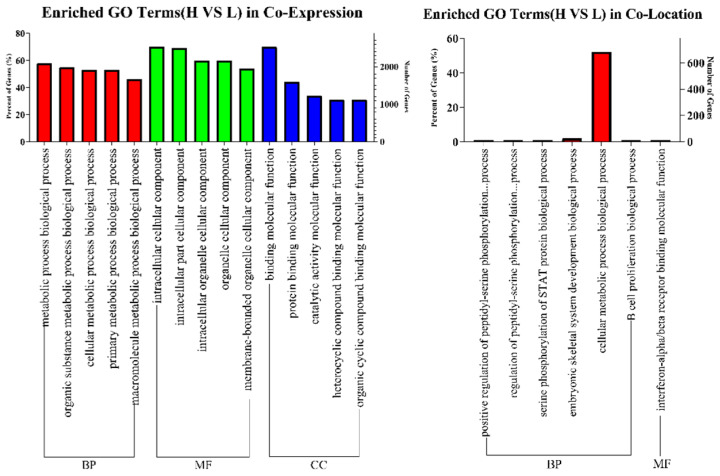
Gene ontology analysis of lncRNA.

**Figure 4:**
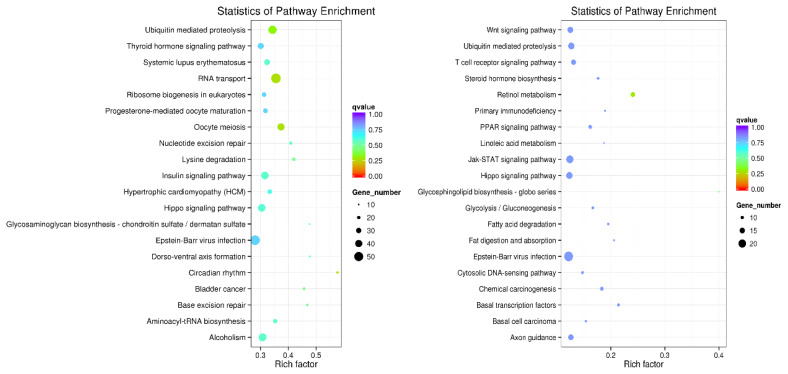
Kyoto Encyclopedia of Genes and Genomes analysis of lncRNA target genes in co-expression and co-location, respectively.

According to the screening criteria for differentially expressed lncRNAs, a total of 493 lncRNAs were found in the RNA-seq data (p<0.05), of which 277 lncRNAs were significantly up-regulated (p<0.05), and 216 lncRNAs were significantly down-regulated (p<0.05) (Figure 2). Based on co-expression target gene analysis, a total of 163 GO terms were significantly enriched in subcutaneous adipose tissue of high and low backfat thickness group (p<0.05), of which 92 terms were involved in BP, 30 terms were involved in MF, and 41 terms were involved in CC. In this study, the top 5 terms with the proportion of gene number were selected and displayed. According to co-location target gene analysis, 7 GO terms were significantly enriched in subcutaneous adipose tissue of the high and low backfat thickness group (p<0.05), of which 6 terms involved BP, 1 term involved MF, and no item involved CC (Figure 3). Based on co-location target gene KEGG pathway analysis, the main signaling pathways include Retinol metabolism and Glycosphingolipid biosynthesis-globo series, etc. The co-expression target gene analysis showed main signaling pathways including RNA transport and Oocyte meiosis, etc. The signaling pathways with the top 20 were shown (Figure 4). Further, we compared lncRNA co-location target genes with co-expression target genes, and found that *ACOX3*, *ILK*, *KCNN4*, *PPAR γ, PRICKLE2* and *RRP8* genes recurred in several pathways (Table 4).

### Results of ordinary polymerase chain reaction amplification

**Figure 5:**
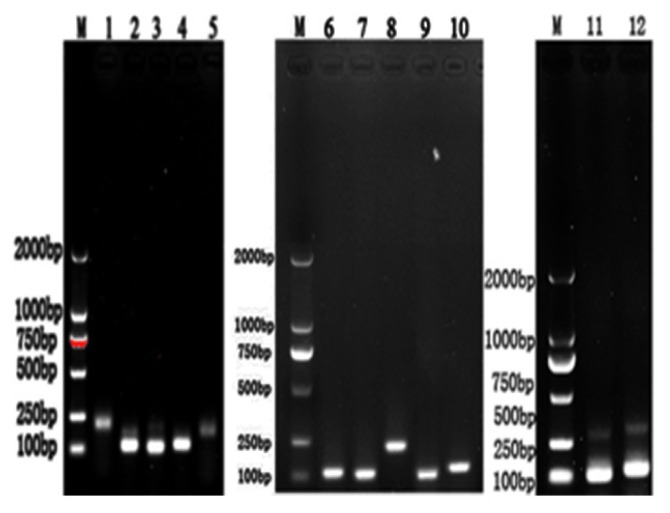
Results of PCR amplification product. M: DM2000 DNA Marker. 1–5: amplification products of *TCONS_00161198*, *TCONS_ 00185937*, *TCONS_00197595*, *TCONS_00191810* and *TCONS_00191803*. 6–12: Amplification products of *PPARγ*, *LPL*, *ACC*, *ATGL*, *GAPDH*, *MOGAT2* and *CSF3R*. PCR, polymerase chain reaction.

lncRNA and target gene fragments were amplified by ordinary PCR and detected by 1.0% agarose gel electrophoresis. The length of PCR amplification products of *TCONS_00161198, TCONS_00185937, TCONS_00197595, TCONS_00191810, TCONS_00191803, PPAR γ, LPL, ACC, ATGL, GAPDH, MOGAT2* and *CSF3R*, was consistent with the expected fragment size, and the electrophoresis bands were clear without trailing or dragging phenomenon, indicating that cDNA and primers could be used for subsequent experiments (Figure 5).

### Validation of real-time fluorescence quantitative polymerase chain reaction

**Figure 6:**
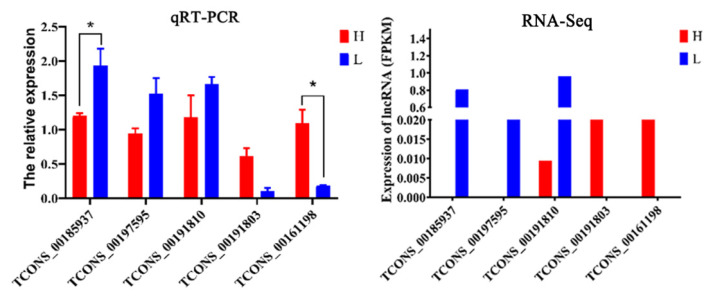
RT-qPCR validation of lncRNA. * Indicates that the difference between the two groups is significant (p<0.05), ** Indicates that the difference between the two groups is extremely significant (p<0.01), the same below. RT-qPCR, reverse transcription quantitative polymerase chain reaction.

According to the results of qRT-PCR and RNA-seq in subcutaneous adipose tissue (Figure 6), the expression of *TCONS_ 00185937*, *TCONS_00197595* and *TCONS_00191810* in low backfat thickness group was higher than those in the high backfat thickness group, and the expression of *TCONS_ 00185937* reached a significant difference (p<0.05). Moreover, the expression of *TCONS_00191803* and *TCONS_00161198* in high backfat thickness group was higher than those in the low backfat thickness group, and the *TCONS_00161198* reached a significant difference. The above results indicated that the expression trend of RT-qPCR was consistent with the RNA-seq, which verified the accuracy and repeatability of the experimental data.

### Correlation analysis of lncRNA expression and phenotypic data of Kele pigs

Correlation analysis results showed that there was a significant negative correlation between backfat thickness and eye muscle area (r = −0.972, p<0.01), and the expressions of *TCONS_ 00161198* and *TCONS_00185937* in subcutaneous adipose tissue were significantly negatively correlated (r = −0.840, p<0.05). The expression of *TCONS_00161198* was significantly positively correlated with backfat thickness (r = 0.946, p<0.01), and negatively correlated with eye muscle area (r = −0.942, p<0.01). The expression of *TCONS_00185937* was significantly negatively correlated with backfat thickness (r = −0.853, p<0.05) (Table 5). Therefore, two lncRNAs *TCONS_ 00161198* and *TCONS_00185937* were selected for subsequent functional verification.

### Bioinformatics analysis of lncRNA

*TCONS_00161198* was located on chromosome 6 of Kele pigs, with 5 exons and a sequence length of 805 bp. *TCONS_ 00185937* was located on chromosome 9, with 2 exons and a sequence length of 846 bp. To investigate the coding ability of lncRNA in biological functions, open reading frames of *TCONS00161198* and *TCONS00185937* were predicted. The results show that there were five open reading frames in *TCONS_0016119*8, and only four open reading frames in *TCONS_00185937*. The specific sequences of open reading frames and encoded amino acids of the two lncRNAs were shown (Table 6). By analyzing the interaction between *TCONS_00161198* and *TCONS_00185937* and their respective target genes, it was found that the 1–39bp of *TCONS_ 00161198* interacts with the 887–930bp of *CSF3R* gene, and the 1–29bp of *TCONS_00185937* interacts with the 356–377bp of *MOGAT2* gene (Table 7).

### Identification of recombinant plasmid

**Figure 7:**
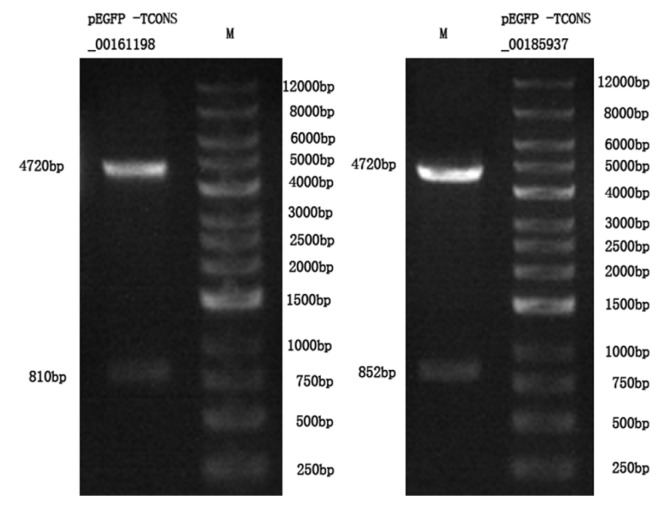
PCR amplification of pEGFP -*TCONS_00161198* and pEGFP -*TCONS_00185937* sequences. PCR, polymerase chain reaction.

To explore the biological functions of *TCONS_00161198* and *TCONS_00185937*. The target gene amplified by PCR was constructed into pEGFP-N3 vector, and the plasmid was extracted. The plasmid was cleaved using HindIII-XhoI enzyme. The strip obtained from pEGFP-*TCONS_00161198* was approximately 810 bp/4,720 bp, which was consistent with the expected size. The strip obtained from pEGFP-*TCONS_ 00185937* was approximately 852 bp/4,720 bp, which was also consistent with the expected size (Figure 7). Further bacterial liquid PCR identification was carried out, and plasmids were extracted from the identified positive bacterial liquid and sequenced by Sangon Bioengineering (Shanghai, China). The sequencing results showed that *TCONS_00161198* and *TCONS_00185937* were completely consistent with the sequences in RNA-Seq.

### Transfected vector into subcutaneous fat precursor cells

**Figure 8:**
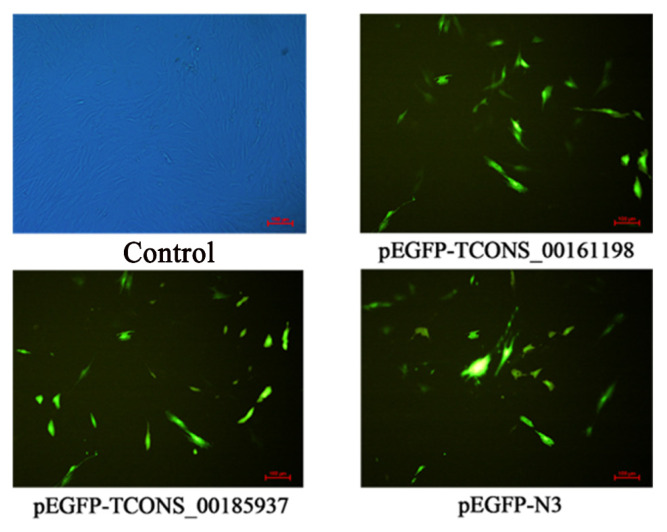
Microscopic examination results of pig subcutaneous fat precursor cells transfected with recombinant plasmids (100×).

In order to study the effect of target lncRNA overexpression on fat deposition in pig subcutaneous fat precursor cells, pEGFP-*TCONS_00161198*, pEGFP-T*CONS_00185937*, and pEGFP-N3 were transfected instantaneously, and the transfection status was observed 36 hours later. The results showed that the transfection group emitted green fluorescence except for the control group, and most of the fluorescence was striped, indicating that pEGFP-*TCONS_00161198*, pEGFP-T*CONS_00185937*, and pEGFP-N3 have been successfully transfected into pig subcutaneous fat precursor cells and can be expressed normally (Figure 8).

### Effect of recombinant vector on gene expression related to fat deposition

**Figure 9:**
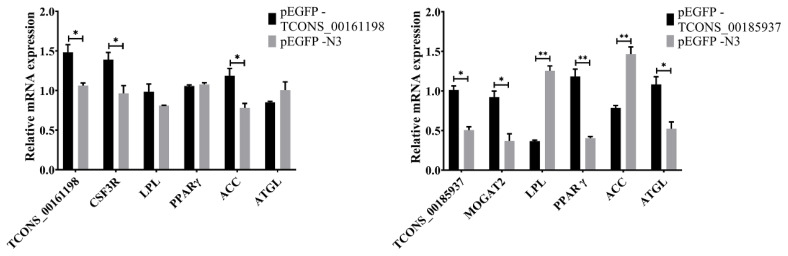
mRNA expression of lncRNA and its target genes, fat deposition-related genes. * p<0.05, ** p<0.01.

To explore the effects of *TCONS_00161198* and *TCONS_ 00185937* on the expression levels of fat deposition related genes in pig subcutaneous fat precursor cells, RT-qPCR was used to detect the expression levels of lncRNA, its target genes and fat deposition related genes after successful transfection for 36 hours. The results showed that the expression levels of *TCONS_00161198* and its target gene *CSF3R* and *ACC* related to fat deposition were significantly higher than those of control group (p<0.05). The expressions of *TCONS_00185937* and its target genes *MOGAT2* and *ATGL* related to fat deposition were significantly higher than those of control group (p<0.05), and the expression level of *PPAR γ* was extremely significantly higher than that of control group (p<0.01), while the expressions of *LPL* and *ACC* were significantly lower than those of control group (p<0.01) (Figure 9).

### Oil red O staining and triglyceride content detection

**Figure 10:**
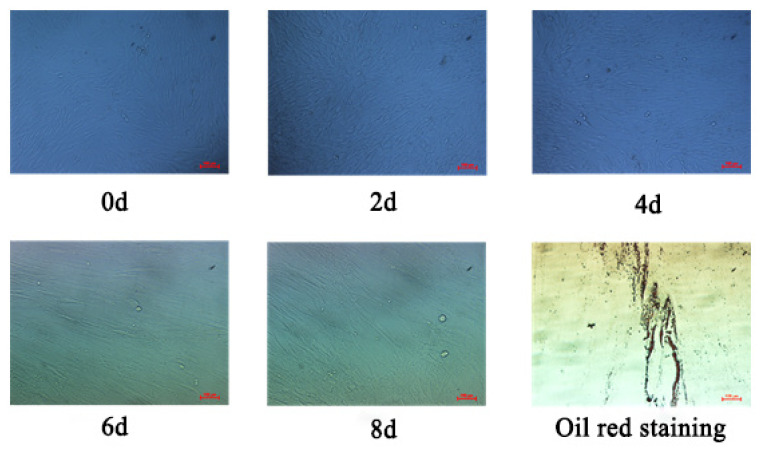
Oil red O staining of pig subcutaneous fat cells. Oil Red O Staining, bar = 100 μm

**Figure 11:**
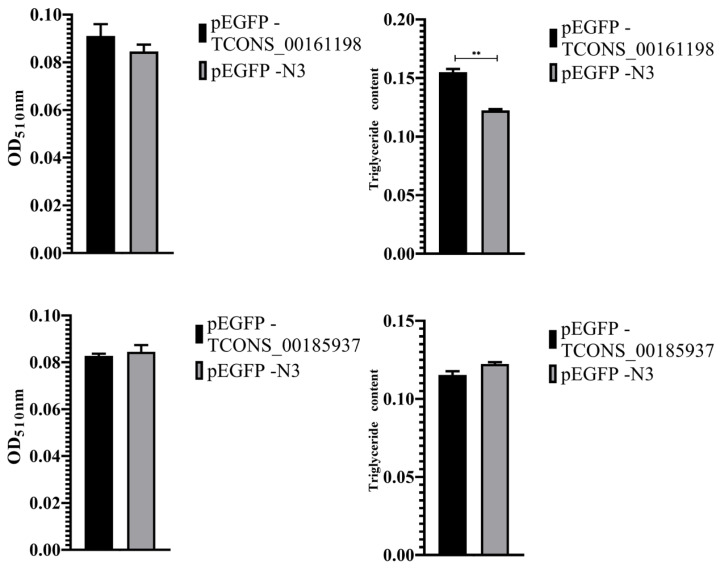
The result of cell extraction and triglyceride content. ** p<0.01.

After 8 days of transfection, oil red O staining was used to identify the fat deposition levels of transfected cells with recombinant plasmid pEGFP-*TCONS_00161198* and pEGFP-*TCONS_00185937* (Figure 10). It was found that elliptical cells began to appear at 2 d induction culture, elliptical cells increased at 4 d, and lipid droplets began to appear, after 6 d induction culture, lipid droplets increased. Triglyceride detection showed that the OD510 light absorption value of pEGFP-*TCONS_00161198* transfection group was higher than that of control group, and the triglyceride content of pEGFP-*TCONS_00161198* transfection group is significantly higher than that of control group (p<0.01). The OD510 value and triglyceride content of pEGFP-*TCONS_00185937* transfection group were lower than control group, but not significantly (Figure 11).

## DISCUSSION

With the advancement of science and technology, lncRNA has been found to play an important role in the biological process, which has attracted special attention [17]. Depending on its location and its specific interaction with DNA, RNA and protein, lncRNA can regulate the assembly and function of chromatin and membranous nucleosomes, alter the stability of cytoplasmic mRNA, and interfere with signaling pathways, thereby regulating the process of fat deposition in animals [20]. Several lncRNAs have been found to be associated with adipogenesis, for example, lncRNA *NEAT1* regulates 3T3-L1 cells [21], lncRNA *ADINR* regulates adipocyte differentiation through transcriptional activation of *C/EPPα* [22], and lncRNA *H19* inhibits adipocyte differentiation of bone marrow mesenchymal stem cells through histone deacetylase [23]. However, the expression pattern of lncRNA associated with Kele pig adipose deposition in subcutaneous adipose tissue has not been reported. Lipoprotein lipase (*LPL*) is a key enzyme involved in fat synthesis, which can hydrolyze triglycerides and regulate fat deposition in animals [24,25]. In this study, the expression of *LPL* gene after transfection of adipose precursor cells with overexpressed vector pEGFP-*TCONS_ 00185937* was significantly lower than that of the control group, which may indicate that *LPL* gene has a negatively regulatory effect on fat deposition in adipose precursor cells. Peroxisome proliferator-activated receptor γ (*PPAR γ*) is an early transcription factor, and proper transcriptional activity of *PPAR γ* is essential for the control of inflammation, tumor and obesity [26]. It can regulate and differentiate undifferentiated mature fat precursor cells into mature fat cells, which are mainly expressed in adipose tissue and participate in important biological processes such as lipid metabolism and differentiation regulation [27]. In this study, the expression of *PPAR γ* gene after transfection of adipose precursor cells with overexpressed vector pEGFP-*TCONS_00185937* was significantly higher than that of the control group, which may indicate that *PPAR γ* gene has a positively regulatory effect on fat deposition in adipose precursor cells. It has been reported that lncRNAs can regulate the expression of adjacent and overlapping coding genes, and can regulate functional genes with protein coding 10–100 kb upstream or downstream of lncRNAs [28]. Therefore, based on the co-location and co-expression relationships of lncRNA, the biological functions of lncRNA were predicted respectively. In this study, significant differentially expressed lncRNAs were first screened, and then co-location target genes and co-expression target genes of lncRNAs were predicted and functional analysis was conducted. The results showed *TCONS_00185937* was significantly enriched in fatty acid degradation signaling pathway, and the target gene *MOGAT2* was enriched in fat digestion and absorption. Fatty acid degradation was a key pathway of fat metabolism, which was involved in the cycle regulation of animal germ cells and affects biological processes [29]. Specifically, the enrichment of *TCONS_00185937* in the fatty acid degradation signaling pathway suggests its potential role in regulating lipid catabolism, which was consistent with the downregulation of *LPL* and *ACC* expression following transfection of pEGFP-*TCONS_00185937* into cells, indicating that *TCONS_ 00185937* may promote lipid breakdown while inhibiting lipid synthesis. *TCONS_00161198* was involved in the activation of the PI3K-Akt signaling pathway, which responds to various cellular stimuli and toxins, regulating essential cellular processes such as cell growth, transcription, translation, proliferation, motility, and glycogen metabolism [30,31]. The enrichment of *TCONS_00161198* in the PI3K-Akt pathway, along with its upregulation of *ACC* and *CSF3R*, indicates its potential role in promoting lipid synthesis and adipocyte proliferation. This finding is supported by the increased triglyceride content observed when transfected into cells of pEGFP-*TCONS_00161198*, further confirming its lipogenic function.

Fat deposition includes the processes of fat synthesis, transport and decomposition. Peroxisome proliferator-activated receptor γ (*PPAR γ*) [32], lipoprotein lipase (*LPL*) [33] and acetyl CoA carboxylase (*ACC*) are key enzymes involved in fat synthesis [34]. Adipose triglyceride lipase (*ATGL*) is a lipolysis enzyme [35], which is one of the important enzymes involved in fat metabolism, and hydrolyzes triglycerides in adipose tissue to regulate the balance between fatty acids and glycerol in cells [36]. In this study, pEGFP-*TCONS_00161198* and pEGFP-*TCONS_00185937* were transfected into pig subcutaneous fat precursor cells, respectively. The expression levels of *TCONS_00161198* and its target gene *CSF3R* and *ACC* were significantly higher than those of control group (p<0.05). The expression levels of *PPAR γ* and *ATGL* were lower than those of control group, but the differences were not significant (p>0.05). However, the expression level of *PPAR γ* in pEGFP-*TCONS_00185937* transfection group was significantly higher than that in control group (p<0.01), and the expression levels of *MOGAT2* and *ATGL* in *TCONS_00185937* and its target genes were significantly higher than that in control group (p<0.05). The expressions of *LPL* and *ACC* were significantly lower than those of control group (p<0.01). Similarly, previous studies have shown that LncIMF2 knockdown inhibits the expression of key enzymes such as *PPAR γ* and *ATGL*, indicating that LncIMF2 can positively regulate adipogenesis, which is similar to the effect observed in *TCONS_00161198* [37]. In addition, two lncRNAs (*TCONS_00012086* and *TCONS_00007245*) related to adipogenesis were identified from Congjiang pigs [38]. According to their target genes and tissue expression profiles, they were found to be associated with increased fat deposition, which was functionally similar to *TCONS_00161198*. These results indicate that lncRNA *TCONS_00161198* and *TCONS_00185937* may regulate their target genes *CSF3R*, *MOGAT2* and *ACC* at the transcriptional level, thereby affecting the fat deposition in the adipose precursor cells in Kele pigs.

Gene function research include gene expression, gene knockout, protein and RNA interaction technology, gene chip and data analysis, yeast identification of target gene function and other molecular biology techniques. In this study, RNA-seq was used to analyze the lncRNA of Kele pigs in high and low backfat thickness groups, including the correlation analysis between the expression level of lncRNA and meat quality traits, the functional enrichment analysis of lncRNA and its target genes GO and KEGG, and other bioinformatics analysis. Then, the lncRNAs related to fat deposition were screened, and the lncRNAs related to backfat thickness were used to verify the gene function using fat precursor cells. The proliferation and differentiation of adipocytes are regulated by a variety of transcription factors, and fat deposition involves a series of complex physiological and biochemical processes [39]. Adipocytes are the main cellular components of adipose tissue, which store excess energy in the form of triglycerides and play a vital role in maintaining the energy balance of cells. The number of adipocytes and the accumulation of triglycerides in adipocytes directly reflect the total fat content of adipose tissue [40]. To further verify the above results, this experiment induced differentiation of pig subcutaneous adipose precursor cells transfected with pEGFP-*TCONS_00161198* and pEGFP-*TCONS_00185937* was carried out. Oil red O staining and triglyceride detection were performed on the induced adipose cells on the 8th day. The results showed that the OD510 absorption value of pEGFP-*TCONS_00161198* transfection group was higher than that of control group, and the triglyceride content of pEGFP-*TCONS_00161198* transfection group was significantly higher than that of control group (p<0.01). The OD510 light absorption value and triglyceride content of pEGFP-*TCONS_00185937* transfection group were lower than those of control group, but there was no significant difference. These results were consistent with the GO and KEGG enrichment analysis, which revealed that *TCONS_00161198* is associated with lipid synthesis pathways (PI3K-Akt signaling), while *TCONS_00185937* is linked to lipid degradation pathways (fatty acid degradation). This functional divergence explains the opposing effects of these lncRNAs on triglyceride accumulation and fat deposition in adipose precursor cells.

In summary, lncRNAs *TCONS_00161198* and *TCONS_ 00185937* displayed distinct and potentially opposing roles in the regulation of fat deposition in Kele pigs. *TCONS_00161198* was implicated in promoting fat deposition, as evidenced by the significantly elevated expression of its target genes, *CSF3R* and *ACC*, which are crucial to fat deposition, and the associated increase in triglyceride content within fat precursor cells transfected with pEGFP-*TCONS_00161198*. This finding suggested that *TCONS_00161198* may have facilitated fat accumulation by upregulating key enzymes in the lipogenesis pathway. In contrast, *TCONS_00185937* appeared to be involved in facilitating fat degradation or maintaining a balance between fat deposition and degradation. The significantly reduced expression of *LPL*, an enzyme crucial for fat synthesis, and the increased expression of *PPAR γ*, which is associated with adipocyte differentiation and fat metabolism, in cells transfected with pEGFP-*TCONS_00185937*. Therefore, our study suggested that these lncRNAs may have antagonistic relationship in the regulatory network of fat deposition, which enhances our understanding of how lncRNAs jointly affect the balance of fat deposition in Kele pigs. In the future, these findings can be leveraged to refine the breeding strategies for Kele pigs through gene editing or the modulation of related gene expression, enabling precise control of fat deposition and enhancing pork quality and breeding efficiency.

## CONCLUSION

We found 493 lncRNAs with significant expression differences, among which several lncRNAs may play a regulatory role in the mechanism related to fat deposition in Kele pigs. Correlation analysis showed that the expression of *TCONS_00161198* and *TCONS_00185937* were significantly negatively correlated, and the expression of *TCONS_00161198* was significantly positively correlated with backfat thickness, and negatively correlated with eye muscle area. Functional validation study found that *TCONS_00161198* and *TCONS_00185937* may regulate their target genes and *ACC* at the transcriptional level, thus affecting the fat deposition in the fat precursor cells, in which *TCONS_00161198* promoted the fat deposition in pig subcutaneous adipocytes. This finding means that we have clarified the regulatory effects of *TCONS_00161198* and *TCONS_00185937* lncRNAs on fat deposition in Kele pigs, providing crucial insights into the underlying mechanisms of adipogenesis.

## Figures and Tables

**Table 1:** Carcass and meat quality traits of Kele pigs

Index		Values	Index	Values
Skin thickness (mm)		4.05±0.82	Drip loss (%)	3.36±2.90
Backfat thickness (mm)		45.98±4.83	Cooked meat (%)	58.21±1.17
Eye muscle area (cm^2^)		24.69±4.13	Moisture (%)	71.73±0.55
Meat color	L*	47.82±5.51	Intramuscular fat content (%)	4.27±1.44
	a*	7.85±1.29		
	b*	4.99±1.40		

**Table 2:** Carcass quality traits of experimental groups of Kele pigs

Index	Group of high backfat thickness	Group of low backfat thickness
Backfat thickness (mm)	50.11±3.33^a^	41.85±0.85^b^
Eye muscle area (cm^2^)	21.65±3.85^a^	27.73±0.20^b^
Intramuscular fat content (%)	4.81±2.06^a^	3.73±0.27^b^
Skin thickness (mm)	4.13±0.22^a^	3.96±1.28^b^

a,b Different lowercase letters indicate significant differences (p<0.05).

**Table 3:** Quality assessment of RNA-seq results

Sample name	Raw reads	Clean reads	Total mapped reads (%)	Q20 (%)	Q30 (%)	GC content (%)
H1	90,659,116	88,345,368	93.50	97.87	94.21	54.85
H2	85,854,704	82,804,864	92.53	98.26	95.25	55.60
H3	89,158,030	86,315,698	92.30	98.27	95.18	56.49
L1	84,911,072	81,667,326	84.36	98.23	95.16	59.51
L2	87,766,298	84,534,016	91.09	98.21	95.07	58.10
L3	87,057,434	84,192,696	92.81	98.34	95.32	57.11

Sample name: Name of sample; Raw reads: the number of sequences in the original data; Clean reads: the number of sequences after the original data was filtered; Total mapped reads: the number of sequences aligned to the genome; Q20: the bases with a base quality value greater than 20 account for the total bases; Q30: the percentage of bases with a base quality value greater than 30 to the total bases. GC content: the percentage of G and C in the available sequence for the four bases.

**Table 4:** List of differentially enriched lncRNA target genes involved in back fat tissues related pathways

Signaling pathway	Target gene of co-location	Target gene of co-expression
Protein digestion and absorption	*SLC8A1*, *KCNN4*^a^, *XPNPEP2*	*COL18A1*, *SLC7A5*, *DPP4*, *CTHRC1*, *KCNN4*^a^, *COLEC10*
PPAR signaling pathway	*ACSBG2*, *RXRA*, *SLC27A6*, *ZBTB45*, *PPAR γ*^a^, *ILK*^a^, *RRP8*^a^, *SLC27A5*	*ILK*^a^, *PPAR γ*^a^, *ACADM*, *SORBS1*, *CPT2*, *MMP3*, *RRP8*^a^, *SLC27A1*, *ACOX3*^a^, *FABP7*, *PTGIS*, *PCK1*
Wnt signaling pathway	*FZD4*, *PLCB4*, *WNT5A*, *PRICKLE2*^a^, *GSK3B*, *PLCB1*, *MAPK10*, *DAAM1*, *SFRP2*, *MSS51*, *PPP3CB*, *MYC*, *TMEM135*, *PRICKLE3*	*ACOX3*^a^, *PEX12*, *PEX1*, *PECR*, *ACOX2*, *HMGCLSFRP1*, *CAMK2D*, *CHD9*, *PRKACB*, *DVL3*, *CSNK1A1*, *CCND3*, *BTRC*, *PRICKLE2*^a^, *RAC1*, *LEF1*, *CAMK2G*, *TCF7L2*, *WNT6*, *PRKCA*, *PLCB3*, *FBXW11*, *APC*, *FOSL1*, *CUL1*, *RAC2*, *MAPK9*

a Ones are found in multiple pathways or at the co-expression and co-location levels.

**Table 5:** Correlation between lncRNA expression and measurement indicators

Project	*TCONS_ 00161198*	*TCONS_ 00185937*	*TCONS_ 00197595*	*TCONS_ 00191810*	*TCONS_ 00191803*	Intramuscular fat	Skin thickness	Backfat thickness	Eye muscle area
*TCONS_00161198*	1								
*TCONS_00185937*	−0.840*	1							
*TCONS_00197595*	−0.655	0.883*	1						
*TCONS_00191810*	−0.370	0.656	0.891*	1					
*TCONS_00191803*	0.848*	−0.895*	−0.623	−0.278	1				
Intramuscular fat	0.744	−0.326	−0.295	−0.166	0.293	1			
Skin thickness	0.108	−0.450	−0.637	−0.751	0.049	−0.061	1		
Backfat thickness	0.946**	−0.853*	−0.717	−0.361	0.863*	0.634	0.163	1	
Eye muscle area	−0.942**	0.735	0.627	0.292	−0.748	−0.786	−0.076	−0.972**	1

* p<0.05,

** p<0.01.

**Table 6:** Prediction of *TCONS_00161198* and *TCONS_00185937* open reading frame

Gene name	Sequence position of ORF (bp)	Coded amino acid
*TCONS_00161198*	1–123	EEESRDQRGQAGAQPASRRLQHGHTEAQKGEVTCSRPPSW
	124–228	ASSRHYGGGAGSLEPGGSCSDHPAAPQKTIRRNP
	229–402	DVQPLWAAKRSGWKPQGGPQHSQQQNGLKNSWERRDGIVSKLQSLLVYSTHSHPAPC
	403–504	ALAMQTVDSLASQNLRSSCGGDRQANNHMTGKS
	505–645	RRIRALEKFRREFPLGLSGLQTQLVIHEDAGSVPGLAHWVKDPALP
*TCONS_00185937*	1–96	SVFLLQIVPLMLYLKSHCQSQSHLDFLLFCN
	97–198	GVLSLPFKFRSMIHFELILGKVYILSLYSLLCV
	457–654	GSSVAMSCGVGHRHGSGPELLWLRYRPAAVAPIRPLAWELPYAMGVALKRKTKAKTKNKKQSGLR
	655–846	RRILSDTVLGSVAAVTNDHNWATSTNRFLCSLTLGGHVGDQFHGIGFQVTGRAGHPPEALGENK

**Table 7:** Prediction target sites of lncRNA and target gene

lncRNA	lncRNA target site (bp)	Target gene name	Target gene site (bp)
*TCONS_00161198*	1–39	*CSF3R*	887–930
*TCONS_00185937*	1–29	*MOGAT2*	356–377

## SUPPLEMENTARY MATERIAL

**Supplement 1**. The volume and reaction condition for reverse transcription.

**Supplement 2**. The primer information of lncRNA and *GAPDH* Gene.

**Supplement 3**. Amplification primer of pEGFP -*TCONS_00161198* and pEGFP -*TCONS_00185937* and some gene.
