# Supplementary material for: Discovery and functional study of lncRNAs associated with fat deposition in Kele pigs based on whole-transcriptome RNA sequencing
Source: Anim Biosci. 2025 Apr 28;38(10):2079–93. doi: 10.5713/ab.24.0900 (PMC12415382; doi:10.5713/ab.24.0900)
Supplement: Supplementary file 1 [file ab-24-0900-Supplementary-1.pdf]

Supplement 1. The volume and reaction condition for reverse transcription

**Reagent**	**Usage amount**	**Reation condition**
RNA	X（50 pg~5μg）	
Olig	1μL	
10n MdNTP mix	2μL	
5×Reaction Buffer	4μL	Vortex mixing, temporary centrifugation<br>42℃ 60 min，75℃ 5 min
Ribo lock Rnase inhibitor (RI)	1μL	
Revert Aid M-Mul VRT (RT)	1μL	
RNase-Free Water	Up to 20μL	
