# Supplementary material for: Discovery and functional study of lncRNAs associated with fat deposition in Kele pigs based on whole-transcriptome RNA sequencing
Source: Anim Biosci. 2025 Apr 28;38(10):2079–93. doi: 10.5713/ab.24.0900 (PMC12415382; doi:10.5713/ab.24.0900)
Supplement: Supplementary file 2 [file ab-24-0900-Supplementary-2.pdf]

Supplement 2. The primer information of lncRNA and GAPDH Gene

**Subject**	**Sequence（5′-3′）**	**Tm(℃)**	**Product Size（bp）**
TCONS_00185937	F：TGGCTGCTGTAACAAACG*ACC*<br>R：CAGTCACTTGGAA*ACC*GATGC	60.8	117
TCONS_00197595	F：ACAGGACATTCAGAAGGCAAGA<br>R：AGGC*ACC*TCCAGGGACAGA	60.8	116
TCONS_00191810	F：TGCTGAATC*ACC*CAGGGAAAG<br>R：GGATGCCAAGTAGGTGCCAAT	60.8	129
TCONS_00191803	F：AATGGA*ACC*TG*ACC*AGC*ACC*T<br>R：GCAGAAATCTATGACAAATAC*ACC*C	58.0	191
TCONS_00161198	F：GGGACAGACAAGCGAATA*ACC*<br>R：CACAGCCATAGCAACTCAGGAT	60.8	234
GAPDH	F：GGTGAAGGTCGGAGTGAACG<br>R：CGTGGGTGGAATCATACTGGA	59.0	152
