# Supplementary material for: Discovery and functional study of lncRNAs associated with fat deposition in Kele pigs based on whole-transcriptome RNA sequencing
Source: Anim Biosci. 2025 Apr 28;38(10):2079–93. doi: 10.5713/ab.24.0900 (PMC12415382; doi:10.5713/ab.24.0900)
Supplement: Supplementary file 3 [file ab-24-0900-Supplementary-3.pdf]

Supplement 3. Amplification primer of pEGFP -TCONS_00161198 and pEGFP -TCONS_00185937 and some gene

primer	Sequence	Tm/°C	Product Length
pEGFP -*TCONS_00161198*	F:CAGATCCGCTAGCGCT*ACC*GGACTCAGATCTCGAGGAAGAGGAGAGCAGAG*ACC*AGCGAGGCCAGGCAGG<br>R:CCCGCGGT*ACC*GTCGACTGCAGAATTCGAAGCTTCTGAACTTTTCCTTTATTTTTATTTTTTGCCTTTTA	58	810 bp
pEGFP -*TCONS_00185937*	F:CAGATCCGCTAGCGCTACCGGACTCAGATCTCGAGTCCGTTTTTCTTTTACAGATTGTGCCTTTGATGCTGTA<br>R:TGGATCCCGGGCCCGCGGTACCGTCGACTGCAGAATTCGAAGCTTCTTATTCTCCCCTAGAGCCTCAGGAGGG	58	852 bp
PPARγ	F:CTCCAAGAAT*ACC*AAAGTGCGA<br>R:CCACAGACTCGGCACTCAAT	52	134 bp
LPL	F: ATCAACAAGGTCAGAGCCAAGA<br>R: TGCCATCCTCAGTCCCAGAA	58	118 bp
ACC	F: GTCCACATGAACAGGCTTCCA<br>R: CCAGTCCGATTCTTGCTCCAC	62	244 bp
ATGL	F: CATCCGTGGCTGCCTGGTGAA<br>R:CCTGGCGGCGAAGTGGGTTAT	62	127 bp
MOGAT2	F: A*ACC*GCAAGGGCTTCGTC<br>R: CCAGGAGCCAGGTAAGTTCTC	60	114 bp
CSF3R	F: CACT*ACC*TCCGCTGCGACTC<br>R: TTGGGACTAAGGGTTCTGG	60	121 bp

Note: The underlined part of the primer is the restriction site
